# Supplementary material for: Safety and efficacy of very low carbohydrate diet in patients with diabetic kidney disease—A randomized controlled trial
Source: PLoS One. 2021 Oct 13;16(10):e0258507. doi: 10.1371/journal.pone.0258507 (PMC8513884; doi:10.1371/journal.pone.0258507)
Supplement: S1 File — (PDF) [file pone.0258507.s002.pdf]

PROTOCOL

TITLE PAGE

Effects of Low Carbohydrate Diet (LCBD) on Weight and Renal Outcome in Patients with Diabetic Kidney Disease (DKD)- A pilot study

Primary Investigator:

Associate Professor Dr Rohana Abdul Ghani

Co-investigators:

Prof Dr Nafeeza bt Hj Mohd Ismail

Dr Aisyah bt Zainordin

Dr Ahmad Azhar bin Abdul Rahim

Cik Fatin Aqilah

Date of document preparation: 8th Oct 2018

Amended date: 10th Jun 2021

Effects of Low Carbohydrate Diet (LCBD) on Weight and Renal Outcome in Patients with Diabetic Kidney Disease (DKD)- A pilot study

Investigator-initiated, single center, randomized, controlled, clinical trial in Type 2 diabetes mellitus patients.

INTRODUCTION

The current population of type 2 diabetes mellitus worldwide is over 200 million and Malaysia contributes to 1.2% of that number[1]. Type 2 diabetes mellitus is a progressive disease associated with debilitating microvascular and macrovascular complications. The prevalence of T2DM in Malaysia has approximately tripled over the last three decades from 6.3% in 1986 to 17.5% of the adult population in 2015 [2]. The prevalence of chronic kidney disease (CKD) in Peninsular Malaysia was high at 9.1% of the adult population in 2011. Among these, 4.2% patients were in CKD stage 1, 2.0% in CKD stage 2, 2.3% in CKD stage 3, 0.2% in CKD stage 4, and 0.4% in CKD stage 5[3]. Diabetes is the leading cause of renal failure for patients commencing dialysis, increasing from 53% of new dialysis patients in 2004 to 61% in 2013[4]. Therefore, diabetic kidney disease (DKD) is a debilitating complication which not only imposes significant health problems but also confers financial burden on affected patients. There has been increasing amount of understanding in the complexity of the relationship between T2DM and obesity. As the prevalence of both conditions continue to demonstrate a parallel rise, the influence of obesity on T2DM is further marked. Thus, this has led to greater emphasis on weight loss in the management of T2DM. More recent anti-diabetic medications including SGLT-2 inhibitors and GLP1 agonists demonstrated greater efficacy in improving glycaemic control and their ability to produce weight reduction. In addition, there has been more interest in the effects of these drugs on retardation of renal disease progression. The mechanism is unclear, either attributed by direct drug effects on renal glomerular-tubular structures, through the Renin-Angiotensin-Aldosterone-System (RAAS), or other pathways. Another plausible explanation is the significant weight loss, which has been shown to have a significant effect of attenuation of renal disease.

Weight reduction programs have long been a complex and tedious treatment plan which has inconsistent, non-duplicable and unpredictable outcomes. Most programs

emphasized on medical nutrition therapy and lifestyle changes. There have been many different dietary plans which share a common goal ie to reduce calorie intake whilst increasing energy expenditure. Few have been successfully reproducible, limited by either patient adherence or modest outcome.

Low carbohydrate diet is a diet plan which stresses on reducing carbohydrate intake to less than 20g daily. Numerous studies have shown that weight loss could be obtained by reduction of calorie intake in either the form of carbohydrate or fat. CKD patients are recommended to consume low protein diet of less than 0.6-0.7g/kg/day with little emphasis on calorie or carbohydrate intake.

This study, thus, aims to evaluate the effects of low carbohydrate and moderate fat (LCBD) in addition to low protein diet on renal disease in patients with DKD.

Objectives

1. To evaluate the effects of LCBD in comparison to low protein diet (LPD) alone on renal outcome in patients with DKD
2. To evaluate the effects of LCBD in comparison to low protein diet (LPD) alone on anthropometric and other metabolic parameters in patients with DKD
3. To determine the associations between renal outcome and metabolic parameters in patients with DKD who received LCBD and standard therapy.

OUTCOME MEASURES

Primary Outcome

1. Proteinuria as assessed by UACR
2. Rise in serum creatinine, reduction in eGFR

Secondary Outcomes

1. Weight loss, blood pressure

2. Metabolic parameters- lipid profile and inflammatory markers including hsCRP, IL-6

HYPOTHESES

LCBD reduces proteinuria and improves renal function compared to standard treatment in patients with DKD.

LCBD causes significant weight loss and improvement in other metabolic parameters compared to standard treatment in patients with DKD.

Study Design

This is an investigator-initiated, single centre, randomized, controlled, clinical trial in Type 2 diabetes mellitus patients, comparing 12-weeks of LCBD compared to standard medical therapy in patients with DKD.

Patient Selection

Recruitment would be conducted at the Endocrinology and Nephrology clinics of UiTM. Patients with Type 2 Diabetes Mellitus undergoing follow-up in these clinics who meet the inclusion and exclusion criteria will be invited to participate in the study.

Inclusion Criteria

1. Diagnosed with Type 2 DM of more than 5 years
2. Diagnosis of stable CKD of more than 6 months
3. HbA1c of 7% - 10.5%
4. Patient age between 40-75 years old
5. Able to sign informed consent

Exclusion Criteria

1. Type 1 DM
2. Frequent hypoglycemia
3. Persistent elevations of serum liver transminase
4. Heart failure (New York Heart Association functional class III–IV), active systemic inflammatory disease, chronic renal failure requiring hemodialysis, active hepatic disease and collagen disease
5. Malignancy
6. Recent hospital admission for acute within the past 3 months
7. Pregnant women, breastfeeding or planning to conceive within the next year

All patients who fulfilled the inclusion and exclusion criteria will be offered to participate in the study and written consent will be obtained.

SAMPLE SIZE CALCULATION

This is a single centre, interventional, randomised, controlled clinical trial. This will be a pilot study involving 20 patients per arm. However, considering a **20% dropout, we would be recruiting 24 patients per arm.**

STATISTICAL ANALYSIS

The SPSS program will be used for the statistical analysis. Differences between before and after intervention will be analyzed by employing one-way ANOVA, following unpaired t-test. A Pearson's correlation analysis would be carried out to determine the correlation of the changes of proteinuria, creatinine, weight loss, and other variables. levels and surrogate endothelial function marker.

STUDY PROTOCOL

Following informed consent, patients would be randomised to either LCBD or low protein Diet (LPD) group.

All recruited patients will be given the standard dietary and exercise advice which will include low protein of 0.6-0.7g/kg/day and low salt diet.

In addition, patients within the LCBD will be given a prescription diet of 20g of carbohydrate daily. This will be supplemented by visual aids on carbohydrate counts of various local food. Patients would be given the option to choose their most appropriate food types which will amount to the carbohydrate count given. Patients on oral anti-diabetic treatment including insulin will advised on titrations of their medications to avoid hypoglycaemia.

The control arm will not be given any additional advice.

All patients will be required to fill in a 3day food diary during their scheduled visits which will be at 6 weeks and 12 weeks.

Clinical assessments include blood sampling which will be done at baseline and at study end.

ULTRASOUND FOR FATTY LIVER

Ultrasound is widely used as a first-line investigation for hepatic steatosis. It provides a qualitative assessment of fatty infiltration of the liver. Grading of diffuse hepatic steatosis can demonstrate the extent of fatty changes in the liver. A senior consultant radiologist who has previous experience in such studies will perform abdominal ultrasound. Each case will be graded as normal, mild fatty liver, moderate fatty liver or severe fatty liver. The liver will be assessed to be normal if the texture is homogeneous, exhibits fine-level echoes, or is minimally hyperechoic or isoechoic compared with normal renal cortex and if there is no posterior attenuation of the ultrasound beam. Mild steatosis is seen as a slight increase in liver echogenicity. In moderate steatosis, visualization of intrahepatic vessels and the diaphragm is slightly impaired, and increased liver echogenicity is present. Severe steatosis is recognized as a marked increase in hepatic echogenicity, poor penetration of the posterior segment of the right lobe of the liver, and poor or no visualization of the hepatic vessels and diaphragm[5].

Dual energy x-ray absorptiometry (DEXA) SCAN[6]

Dual energy x-ray absorptiometry (DEXA) was once used for determining bone density however it has now evolved into a technique for also estimating body composition. DEXA can measure bone mineral content (BMC), bone mineral density (BMD), fat-free mass (FFM), and can also provide estimates of percent body fat. It is widely regarded as one of the most proven and reliable technologies for measuring body composition. DEXA works by measuring the body's absorbance of x-rays at two different energies. Fat, bone mineral, and fat-free soft tissue have different absorption properties. The amount of radiation exposure during a DEXA scan varies based on the region of the body scanned and is much lower than standard plain radiography examinations. DEXA scan is less than two days' exposure to natural background radiation (NBR). In comparison, a plain radiograph of the chest is equivalent to approximately three days of exposure to NBR, and a transatlantic flight is approximately a week's exposure to NBR. Improved technology has dramatically decreased scan times to 5-10 minutes making this method convenient and non-invasive. DEXA allows accurate and comprehensive measurements of total body fat percentage, and also segmental body fat distribution in regions such as arms, legs, waist and hips. Another advantage of DEXA is it can measure the visceral fat content in the waist region. Visceral fat is of particular concern as it is a key player in many

preventable diseases. It is extremely valuable in research as it can be used to obtain an initial body composition assessment and to understand how body is changing over time, that is, how much lean and fat tissue changes over time therefore measuring the impact of training or dietary programs. The scan is a quick and painless procedure whereby the patient lies on the back of the DEXA table. No special preparation is needed before the scan. Analysis of the results is done by the DEXA computer software.

References:

1. Feisul MI, Azmi S. (Eds). National Diabetes Registry Report, Volume 1, 2009-2012. Kuala Lumpur; Ministry of Health Malaysia; 2013 Jul.
2. National Health and Morbidity Survey 2015 (NHMS 2015). Ministry of Health, Kuala Lumpur, Malaysia(2015)
3. L.S. Hooi, L.M. Ong, G. Ahmad, *et al.*A population-based study measuring the prevalence of chronic kidney disease among adults in West Malaysia. Kidney Int, 84 (2013), pp. 1034-1040.
4. B. Goh, L. Ong, Y. LimTwenty First Report of the Malaysian Dialysis and Transplant 2013. Malaysian Society of Nephrology, Kuala Lumpur, Malaysia (2014)
5. Strauss S, Gavish E, Gottlieb P, KatsnelsonL . Inter-observer and Intra-observer Variability in the Sonographic Assessment of Fatty Liver. AJR:189, December 2007 W320–W323)
6. Australian Radiation Protection and Nuclear Safety Agency. Ionizing Radiation and Health. Updated 3 December 2003. *https://www.legislation.gov.au/Details/F2019C00829*

FLOWCHART

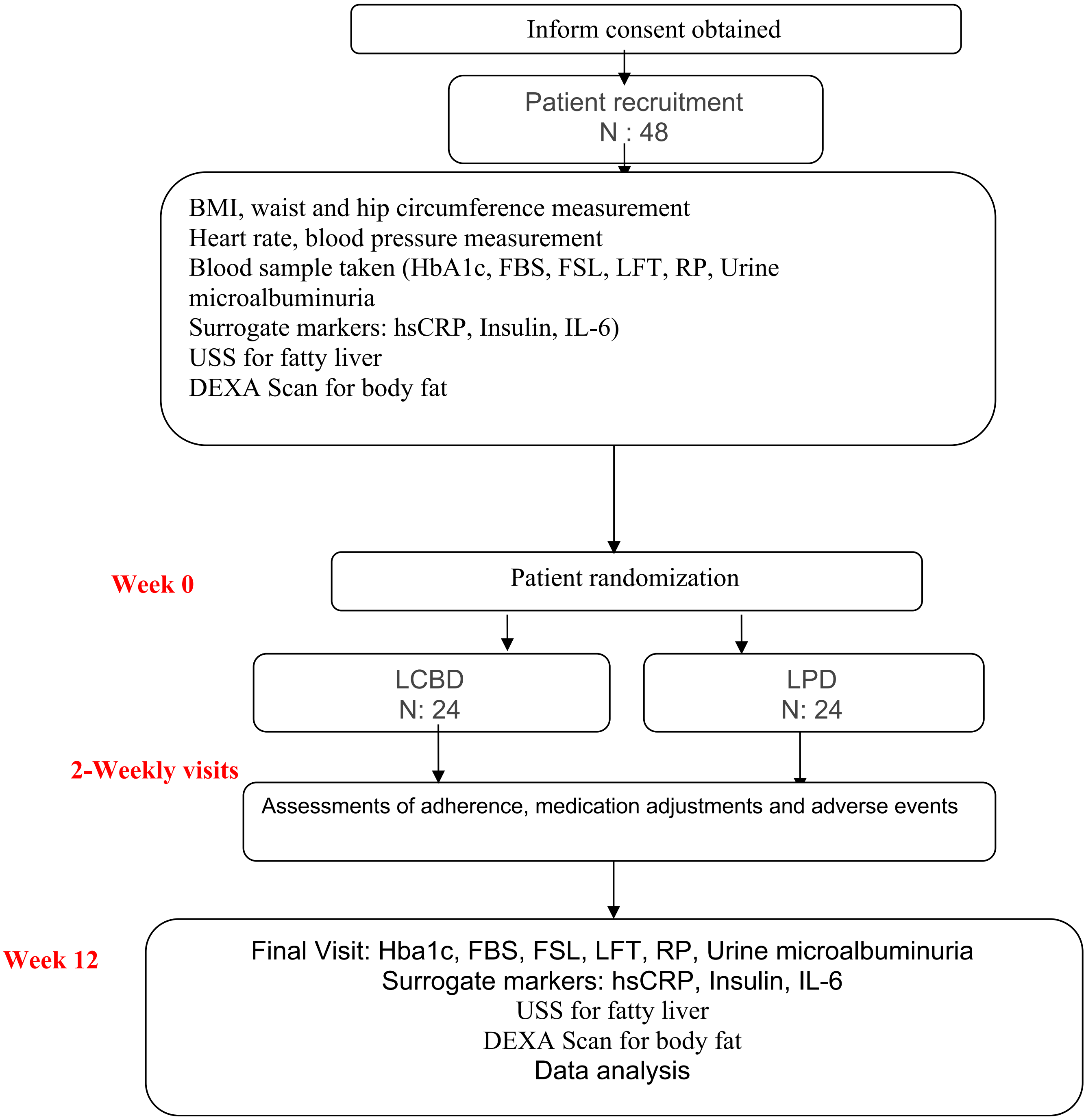
Inform consent obtained
Patient recruitment
N : 48
BMI, waist and hip circumference measurement
Heart rate, blood pressure measurement
Blood sample taken (HbA1c, FBS, FSL, LFT, RP, Urine microalbuminuria
Surrogate markers: hsCRP, Insulin, IL-6)
USS for fatty liver
DEXA Scan for body fat
Week 0
Patient randomization
LCBD
N: 24
LPD
N: 24
2-Weekly visits
Assessments of adherence, medication adjustments and adverse events
Week 12
Final Visit: Hba1c, FBS, FSL, LFT, RP, Urine microalbuminuria
Surrogate markers: hsCRP, Insulin, IL-6
USS for fatty liver
DEXA Scan for body fat
Data analysis

Effects of Low Carbohydrate Diet (LCBD) on Weight and Renal Outcome in Patients with Diabetic Kidney Disease (DKD)- A pilot study
Revised Budget

No	Item	Price per item (RM)	Quantity	Total (RM)
1	Fasting plasma glucose	15	96	1,440
2	HbA1c	25	96	2,400
3	Lipid profile	15	96	1,440
4	Liver profile	15	96	1,440
5	Renal profile	15	96	1,440
6	UPCI	15	96	1,440
6	ELISA kits for surrogate markers hsCRP	2000/kit	2	4,000
7	ELISA kits for IL-6	3000/kit	2	6,000
8	Insulin (for 100 tests)	2,350	1	2,350
9	Glucometer and strips	200	48	9,600
10	Patient reimbursement	50/ visit	50 x 9 x 24 = 50 x 7 x 24	10,800 + 8,400 = 19,200
11	USS (Fatty liver)	50	96	4,800
12	Dexa	190	96	19,200
10	Stationery + Visual Aids	50/patient	48	2,400
			TOTAL	76,950

Gaant Chart

See Appendix A

Key Milestones

Milestone	Timeline	Milestone description
**Ethics Approval**	Sept 2018	Starting of recruitment
**Patient Recruitment**	Dec 2018	50% recruitment
	March 2019	100% recruitment
**Patient randomisation**	Dec 2018<br>Blood sampling and other measurements	50%
	April 2019	100% randomised
**Data Compilation**	Jul 2019	100% data compilation
**Data Analysis and interpretation**	Sept 2019	Completed data collection and performing Statistical analysis
**Report Writing**	Dec 2019	Completed report writing
**Submission report**	Feb 2020	Submission completed

Appendix A

Research Activities	April 2018	May 2018	Jul 2018	Aug 2018	Sep 2018	Oct 2018	Nov 2018	Dec 2018	Jan 2019	Feb 2019	Mar 2019	Apr 2019	May 2019	Jun 2019	Jul 2019	Aug 2019	Sep 2019	Oct 2019	Nov 2019	Dec 2019	Jan 2020	Feb 2020
Literature Review																						
Proposal Development																						
Proposal Defense and Approval																						
Ethical Approval																						
Patient Recruitment and randomisation																						
Data Collection																						
Data Entry and Analysis																						
Report Writing																						
Submission of Report																						

Patient Information Sheet

Dear Sir / Madam,
Thank you for your consideration to participate in this trial. Enclosed is a brief introduction and outline of the study.

What is the name of the study?
Effects of Low Carbohydrate Diet (LCBD) on Weight and Renal Outcome in Patients with Diabetic Kidney Disease (DKD)- A pilot study

Why are we doing this study?
Diabetes mellitus is a progressive disease and associated with many complications. Kidney failure is one of the serious and debilitating disease. Besides good glycaemic control, previous studies have shown reducing protein in your diet and weight loss may help slow the progression of kidney disease. Weight loss is difficult but reducing calori intake, particularly carbohydrate in your diet, may produce significant results.
Thus, this study aims to determine the effects of low carbohydrate diet on renal outcome in patients with DKD.

Why have I been chosen?
You are a patient with diabetes with mild to moderate diabetic kidney disease.

Do I have to take part?
It is up to you whether or not to take part. If you decide to take part, you will be given this information sheet to keep and be asked to sign a consent form.

What will happen to me if I take part?
If you agree to participate in the study, you will undergo some routine blood tests. You will be given standard dietary and exercise advice. You will be then randomised into 2 groups. If you are included in the intervention group you will be asked to reduce your carbohydrate content in your diet an amount of 20g/ day. You will be given clear instructions on how you would be able to achieve this. If you are in the control group you will not receive any additional advice. You will bemonitored in the next 12 weeks, at the end of which another battery of blood tests will be performed at no added cost. You will be given a limited supply of glucometer and strips throughout the study period.

What do I have to do?
You are required to adhere to your dietary prescription and provide a 3-day food diary for the required study visits. You are also required to perform regular blood sugar monitoring at home. You are also required to undergo an *ultrasound test* to detect fatty liver. This test is completely harmless and will not cause any pain to you. A *bone densitometry* test will also be performed to look at your body fat. This test has the radiation of 1/10th of a normal chest-XRay. These investigations will be conducted without any cost to you.

What kind of monitoring is offered during the study?
Blood test will be obtained at baseline (before start of trial) to assess your kidney function, liver function, fasting lipids, glucose, insulin, HbA1c and 2 other inflammatory markers ie hsCRP and IL-6.

What are the possible disadvantages and risks of taking part?
You might experience mild discomfort during blood taking. Other than that, the dietary prescriptions may result in some symptoms of lethargy with possibility of episodes of hypoglycaemia. Thus, the need for regular home blood glucose monitoring (HBGM).

<u>What are the possible benefits of taking part?</u>
You will be given appropriate dietary advise, which may result in significant weight loss and subsequently improve your diabetes control. You will obtain an USS for fatty liver and a bone densitometry for free.

<u>What if new information becomes available?</u>
We will let you know as soon as possible and re-obtain your consent (should you agree to continue) after discussing with you any new findings appear during the trial.

<u>What happens when the research study is terminated before the study end date?</u>
We will notify you immediately and provide you with proper follow-up.

<u>Will my taking part of this study be kept confidential?</u>
Yes. Your name will never be released to the public. If and when a publication of this study is submitted/published, your participation in this study will remain anonymous.
Thank you for your interest and your time. We will be happy to answer any questions that you have.
If you have any queries please do not hesistate to call our clinic at the following numbers: +60361265131

Prof Dr Rohana Abdul Ghani
Prof Dr Nafeeza bt Hj Mohd Ismail
Dr Aisyah bt Zainordin
Dr Ahmad Azhar bin Abdul Rahim
Cik Fatin Aqilah

Confidentiality
Your medical information will be kept confidential by the researcher and will not be disclosed unless it is required by law.
By signing this agreement form, you authorize the records research, analysis and data usage of this study.

Consent Form

Effects of Low Carbohydrate Diet (LCBD) in Patients with Diabetic Kidney Disease (DKD)

To become a subject in the research, you or your legal guardian is advised to sign this Consent Form. I herewith confirm that I have met the requirement of age and am capable of acting on behalf of myself /* as a legal guardian as follows:

1. I understand the nature and scope of the research being undertaken.
2. I have read and understood all the terms and conditions of my participation in the research.
3. All my questions relating to this research and my participation therein have been answered to my satisfaction.
4. I voluntarily agree to take part in this research, to follow the study procedures and to provide all necessary information to the investigators as requested.
5. I may at any time choose to withdraw from this research without giving reasons.
6. I have received a copy of the Subjects Information Sheet and Consent Form.
7. Except for damages resulting from negligent or malicious conduct of the researcher(s), I hereby release and discharge UiTM and all participating researchers from all liability associated with, arising out of, or related to my participation and agree to hold them harmless from any harm or loss that may be incurred by me due to my participation in the research.

____

Name of Subject/Legal Guardian **Signature**

____

I.C No **Date**

____

Name of Witness **Signature**

____

I.C No **Date**

____

Name of Consent Taker **Signature**

____

I.C No **Date**
