# Supplementary material for: Safety and efficacy of very low carbohydrate diet in patients with diabetic kidney disease—A randomized controlled trial
Source: PLoS One. 2021 Oct 13;16(10):e0258507. doi: 10.1371/journal.pone.0258507 (PMC8513884; doi:10.1371/journal.pone.0258507)
Supplement: Attachment — Submitted filename: Response Letter 2 for Reviewers VLCBD.docx [file pone.0258507.s005.docx]

26^th^ Aug 2021

Editor

PLOS ONE

Dear Sir,

**Revisions for: Safety and Efficacy of Very Low Carbohydrate Diet in Patients with Diabetic Kidney Disease (PONE-D-21-17598)**

Thank you for your prompt response and further comments and suggestions. I hereby provide the details of the amendments as required.

Academic Editor:

The reviewers are mostly satisfied with the revised manuscript. Please comment on what seems to be common underreporting of energy intake in the discussion, as per Reviewer 1. If food was not provided to the study participants, please add as a limitation as well as the dietary reporting using 3-days diet diaries.

*Thank you so much for these suggestions. The points have been included as suggested.*

Reviewer #1: The authors have addressed my concerns and the manuscript has improved. However, reported baseline calorie intake is extremely surprising, being very hypocaloric in patients with obesity. I suggest to review records once again and elaborate on this aspect which seems extremely unlikely to be true.

*Thank you for the comments. We have reviewed the records again and confirm the findings. However, we acknowledge the points raised and have included this in the discussion and limitation accordingly.*

*This statement has been added. “With regards to the baseline macronutrients intakes, the notably low baseline calorie consumption could probably be due to considerable under-reporting and under-estimation, both intentionally and unintentionally, as similarly reported by a previous study [25].”
References have been adjusted accordingly.*

Moreover, English language needs extensive revision especially in the newly added parts.

*We have sent the manuscript for proof reading. The text score was 82%. Corrections have been made accordingly.*

Thank you again for your suggestions and comments. We look forward to your positive review.

Sincerely yours,

**Corresponding author:**

**Prof Dr Rohana Abdul Ghani**

Consultant Endocrinologist
Endocrine Unit

Department of Internal Medicine

Faculty of Medicine

Universiti Teknologi MARA (UiTM),

Selangor, Malaysia.
